# Mortality Predictors in Covid-19 Positive Patients with Fractures: A Systematic Review

**DOI:** 10.30476/BEAT.2021.87742

**Published:** 2021-04

**Authors:** Arvind Kumar, Yawar Haider, Jigyasa Passey, Rizwan Khan, Sahil Gaba, Mukesh Kumar

**Affiliations:** 1 *Department of Orthopaedics, Hamdard Institute of Medical Sciences and Research, New Delhi, India*; 2 *Department of Anatomy, Maulana Azad Medical College, New Delhi, India*; 3 *Department of Orthopaedics Surgery, Woodend Hospital, Aberdeen, Complete Abbreviation (GBR), City, Country*

**Keywords:** COVID 19, Fractures, Mortality, Predictors, Risk Factors

## Abstract

**Objective:**

To analyze the factors associated with mortality in fracture patients with concomitant COVID-19 infection based on the available published data.

**Methods:**

Keywords such as “fracture” and “COVID or COVID-19” were searched through three major databases includes PubMed, EMBASE, and Google Scholar. Selection criteria were all published reports providing the mortality related information of COVID-19 positive fracture patients. Published papers containing mortality data of COVID-19 positive fracture patients were considered for qualitative review. For meta-analysis, the presenting individual’s data were considered to study the different parameters association with mortality.

**Results:**

The rate of mean mortality in COVID-19 positive fracture patients was 34%, and 91.7% of patients had hip fractures. Older age and hip fractures had a significant association with higher mortality rates in COVID-19 positive fracture patients.

**Conclusion:**

The mortality rates are considerably higher in COVID-19 positive patients with fractures compared to COVID-19 positive patients without fractures and to the COVID-19 negative fracture patients. Early surgical intervention should be preferred in hip fractures among COVID-19 positive patients for general stabilization and improved respiratory function. Older age and hip fractures are the main predictors of mortality in these patients.

## Introduction

The COVID-19 pandemic has become a significant healthcare issue worldwide. The negative impact has created different domains in patient’s management by considering the high mortality rates [1]. The disease has affected more than 18 million people since its emergence in late 2019 in Wuhan province of China. Little is known about the fracture management in COVID-19 positive patients because of its progression and mortality characteristics from this short period, while several reports have been available regarding the disease. The available evidence comes from scattered reports which suggest a higher risk of perioperative mortality in COVID-19 positive patients [2] and other studies suggesting an uneventful perioperative period [3, 4]. Some reports had also suggested to delay the operative management in fractures until the active phase of the disease is over or the patient has become COVID-19 negative [5, 6]. However, a delay in operative management for lower limb fractures is equivalent to a bedridden status in elderly patients and it is a major predictor of mortality [7]. 

Additionally, the active phase duration and progression of disease is difficult to predict in fractured patients because the fracture severity and extent can itself bring a change to the level of the inflammatory markers and their levels may further change due to fracture-related complications. The currently available evidence suggests a role of cardiometabolic disease and end-organ failures in non-fractured COVID-19 affected patients to be the chief predictors of mortality [8]. However, there is very limited regarding the predictors of mortality in fracture affected COVID-19 positive patients. Knowledge of these predictors can help to plan the fracture’s management and most importantly to predict the prognosis in fracture patients especially the elderly patients who are at the risk of further deterioration due to fracture-related complications. The current systematic review aims to review the available evidence for comprehensively analysis the mortality predictors in COVID-19 positive fracture patients.

## Materials and Methods

In accordance with the preferred reporting items for systematic reviews and meta-analyses, two of the present study authors independently searched through Pubmed, Google Scholar and Embase on 27^th^ June 2020 by Complete abbreviation (PRISMA) guidelines. We used the key terms such as “Fracture” and “COVID or COVID-19”. These two authors were screened the titles and abstracts of the search results for their relevance to the mortality in COVID-19 affected patients presenting with fractures. The full texts of the included studies were analyzed by the two authors. Those articles were included in the study which were presented the mortality-related information of COVID-19 affected fracture patients. All of the included articles were considered for the qualitative review. Those articles were examined for additional quantitative analysis and they were provided the mortality details of individual patients. A secondary manual search was performed for additional articles by scrutinizing bibliographies of publications identified. We excluded those studies with no description of COVID-19 affected fracture patients and those merely discussing fracture management during the COVID-19 pandemic. 

*Statistical Analysis*

The quantifiable parameters related to individual patients were recorded and the data were pooled in an Excel sheet for statistical analysis. The parameters were broadly classified among the following categories: patient’s characteristics and comorbidities, fracture characteristics, inflammatory markers and surgery-related parameters. Review Manager (Rev Man, Version 5.3. Copenhagen: The Nordic Cochrane Centre, The Cochrane Collaboration, 2014) was used to perform a meta-analysis of the studies providing quantitative data related to mortality in COVID-19 infected fracture patients. The dichotomous data were analyzed using the combined estimates of the odds ratios (OR) correlating the variation of the above stated parameters to the mortality in COVID-19 infected fracture patients. The continuous data were analyzed using weighted mean differences (WMD) and 95% confidence intervals (95% CI) of the above stated parameters divided into mortality and survival groups. For each of the analyses, the fixed-effect model and heterogeneity (I^2^) of less than 50% were aimed for, and studies lying grossly outside the funnel plot were excluded for individual analysis. A *p*-value of <0.05 indicated statistical significance.

## Results

**Fig. 1:**
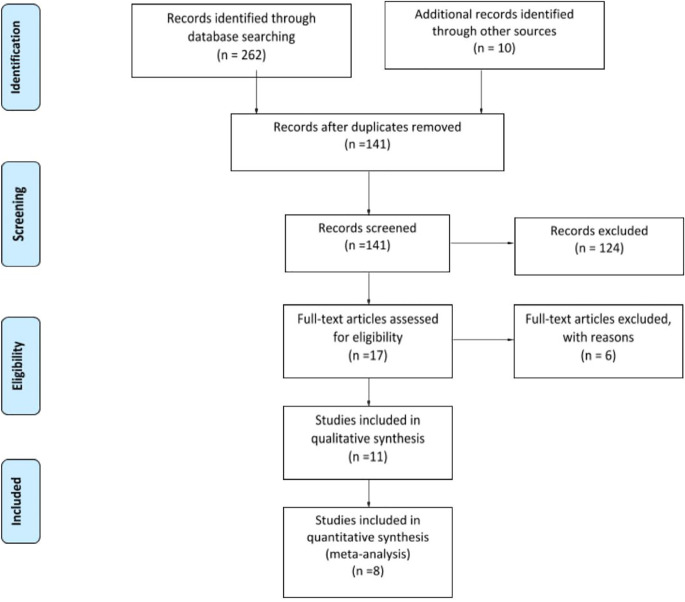
PRISMA Flow Diagram of studies for the current systematic review

The search yielded 55 articles on PubMed, 75 on EMBASE, and 132 on Google scholar. After excluding the duplicate ones, an initial screening of the titles revealed seventeen articles that discussed the COVID-19 management affected fracture patients (Figure 1). Eleven out of the seventeen selected articles provided the mortality related information of COVID-19 infected fracture patients, and those were included for the qualitative review. Eight articles provided mortality related information of individual COVID-19 infected fracture patients, and those were considered for quantitative analysis. The secondary manual search found ten articles but those were already added from the database results.

*Qualitative Review*

The COVID-19 infection was found to increase morbidity and mortality in fracture patients. The mortality data comes from small cohort observational studies and case series of variable sample sizes. Among the eleven studies included in the qualitative synthesis, a total of 150 fracture patients were managed with concomitant COVID-19 infection. The overall mortality was high (mean = 34%) with maximum deaths reported in patients with hip or proximal femoral fractures. In COVID-19 positive patients with hip fractures, the mortality was 56%. Interestingly, 91.7% of the fractures among the reviewed studies belonged to the proximal femoral fractures in elderly patients suggesting a low energy trauma profile of the presenting fractures. The mean age was 77.32 years suggesting an overall elderly cohort of patients. The detailed observations from the reviewed studies have been provided in Table 1.

**Table 1:** Review of the studies presenting the mortality rates in COVID-19 positive fracture patients

**Authors **	**Region**	**Sample size of COVID positive fracture patients**	**Fracture type**	**Mean age of COVID positive patients ( in years)**	**Mortality in COVID positive patients with fractures**	**Mortality in COVID -ve patients with fractures**	**Significant associations with mortality **
Cheung ZB *et al*., [4]	USA (New York)	10	10 proximal femur fractures	79.7	1 (10%)	-	-
Catellani F *et al*., [9]	Italy	16	16 Proximal femur	85	7 (43.7%)	-	-
Muñoz Vives JM *et al*., [10]	Spain	23	23 Proximal femur	85.3	7 (30.4%)	6 (5.3%)	Non-operative management, Cough on presentation, higher ASA grade, lobar consolidation on chest radiograph
Mi B *et al*., [11]	China (Wuhan)	10	7 Proximal femur, 2 spine, 1 forearm	68.4 years	4 (40%)	-	Old age
LeBrun DG *et al*., [12]	USA (New York)	9	9 Proximal femur	85	5 (56%)	4%	Higher ASA grade
Egol KA *et al*., [13]	USA (New York)	31	31 proximal femur	C+: 82.4 Cs: 80.6	11 (35.5%)	6 (5.6%)	-
Maniscalco P *et al*., [14]	Italy	32	32 Proximal femoral	81.1	9 (29.1%)	2 (22.2%)	-
Rizkallah M *et al*., [15]	France	12	4 proximal femur, 3 Hip prosthesis dislocation, 2 ankle, 1 proximal humerus, 2 spine	78.1	5 (41.6%)	-	Proximal femoral fractures
Hernigou J *et al*., [16]	Belgium	4	3 proximal femur, 1 lower limb fracture	NA	2 (50%)	None	Older age
Garcia-Portabella M *et al*., [17]	Spain	1	1 humerus	43	None	None	-
Song SK *et al*., [18]	South Korea	2	2 proximal femur	82	None	-	-

The elderly patients with lower limb fractures carry a risk of chest deterioration if not mobilized early. The accompanying COVID-19 illness can pose a major challenge in the operative management of these patients due to either preoperative COVID-19 pneumonia or postoperative respiratory deterioration. Catellani *et al*., [9] suggested an improvement in respiratory function with early mobilization, and therefore, early operative intervention should be sought whenever feasible. They proceeded with early surgery in patients with a pO2 of >90% and a body temperature of <38°C. Severe pneumonia and respiratory insufficiency were considered as contraindications to surgery. Supplementary oxygen and arrangement for mechanical ventilation should, therefore, be available to manage the respiratory symptoms. Hypostatic pneumonia can develop in elderly patients with lower limb fractures and may create difficulty in assessing the COVID-19 pneumonia. Despite their high mortality rates, Muñoz Vives *et al*., [10] advocated for early surgical treatment in patients with clinical features of COVID-19 infection as those patients could further improve following early mobilization. Their mortality rates were significantly high in non-operatively managed proximal femoral fractures (mortality rate=67%) compared to the operatively managed patients (mortality rate=4%) with concomitant COVID-19 infection. Similarly, Mi *et al*., [11] advocated the high rates of mortality in non-operatively managed patients for early surgical management in COVID-19 affected patients with hip fractures. Three out of 4 deaths in their series were recorded in patients that underwent non-operative management while 70% of the included patients were symptomatic due to COVID-19 illness.

Cheung *et al*., [4] observed the least mortality of 10% despite involving elderly COVID-19 affected patients with proximal femoral fractures. Their mean age was 79.7 years. However, the important take away points from their observations were that were asymptomatic of 80% involved patients, and all were operated within 48 hours. Additionally, all patients undergoing testing had at least one marked raised above normal levels for inflammatory markers preoperatively, suggesting that early operative intervention can be helpful in such patients as well. LeBrun *et al*., [12] observed an increased need for oxygen supplementation in COVID-19 affected hip fracture patients due to hypoxia (44%) compared to COVID-19 negative patients with hip fractures (6%). Egol *et al*., [13] observed a significant increase in early fracture-related mortality from 3% in the year 2019 to 12.3% in the year 2020. The mortality rates were significantly high in COVID-19 confirmed (37.3%) and COVID-19 suspected (7.1%) patients with hip fractures compared to COVID-19 negative patients with hip fractures (5.6%). Additionally, the COVID-19 infected patients with hip fractures were found to have a significantly longer hospital stay, higher complication rates, and greater incidence of mechanical ventilation postoperatively. Likewise, Maniscalco *et al*., [14] observed an increased rate of deaths in COVID-19 patients with hip fractures. Rizkallah *et al*., [15] compared the mortality rates of COVID-19 infected fracture patients to the non-fracture COVID-19 positive patients. The mortality rates were significantly high in fractured COVID-19 positive patients (41.6%) compared to non fractured COVID-19 positive patients (8.33%). The most common cause of death in the reviewed studies was respiratory failure as a result of COVID-19 pneumonia. Cardiac and multiple organ failures were the second and third most common causes of death, respectively. It has been advocated that the COVID-19 illness can exacerbate preexisting comorbidities. However, respiratory failure remains the major cause of death irrespective of pre-existing illnesses. Therefore, early arrangements of oxygen supplementation and mechanical ventilation should be made prior to handle the COVID-19 affected fracture patients. The incidence and proportions of the other fractures, especially upper limb fractures and high energy fractures in COVID-19 positive patients were very low. The lockdown situation and reduced population flow could possibly have contributed to the reduction of high energy trauma around the world which mostly occurs in road traffic accidents. The incidence of the upper limb fractures could be low because of generalized preference to delay surgery of these fractures in COVID-19 positive patients. These fractures seldom require immediate in-patient care in pandemic situations compared to the lower limb fractures in elderly population, where the risk of life-threatening complications is high. The most common surgical management for the proximal femur fractures other than the femoral neck fractures was cephalomedullary nail fixation and hemiarthroplasty for femoral neck fractures. The detailed influence of various surgical and non-surgical factors has been analyzed in quantitative analysis.

*Quantitative Analysis (Meta-Analysis)*

Eight articles were considered for the quantitative synthesis as they provided information of individual COVID-19 positive fracture patients [4, 9-12, 15, 17, 18]. Individual data of sixty-four patients were pooled for the analysis (Tables 2 and 3).

**Table 2:** Dichotomous comparison of individual categorical variables for mortality in COVID-19 infected patients with fractures.

**Variable**	**Odds ratio **	**95% Confidence interval**	**Total number of cases**	***p*** **-value**	**Remarks **
Gender	1.40	0.39, 5.02	64	0.61	Statistically insignificant
CNS disorders	1.69	0.38, 7.44	45	0.49	Statistically insignificant
Diabetes Mellitus	0.63	0.15, 2.61	45	0.53	Statistically insignificant
Hypertension	0.29	0.07, 1.24	45	0.10	Statistically insignificant
Cardiac disorders	0.26	0.05, 1.46	45	0.13	Statistically insignificant
Renal disorders	0.52	0.11, 2.61	45	0.43	Statistically insignificant
Respiratory disorders	3.56	0.39, 32.83	45	0.26	Statistically insignificant
Bowel disorders	2.45	0.26, 23.33	45	0.43	Statistically insignificant
Hip fractures vs. non-hip fractures	9.76	1.26, 75.72	61	0.03	*p*<0.05 (statistically significant)
Lower limb fractures vs. upper limb fractures	2.94	0.25, 33.90	59	0.39	Statistically insignificant
Operative management vs. non operative management	0.90	0.21, 3.96	64	0.89	Statistically insignificant
Hemiarthroplasty vs. cephalomedullary nailing	1.23	0.22, 6.98	28	0.82	Statistically insignificant

**Table 3:** Comparison of individual continuous variables for mortality in COVID-19 infected patients with fractures

**Variable**	**Mean difference**	**95% Confidence interval**	**Total number of cases**	***p*** **-value**	**Remarks**
Age	4.30	0.44, 8.15	48	0.0007	*p*<0.05 (statistically significant)
Total number of comorbidities	-0.66	-1.34, 0.01	45	0.06	Statistically insignificant
Delay in surgery	0.15	-4.14, 4.44	24	0.95	Statistically insignificant
TLC	-4.66	-11.85, 2.53	30	0.20	Statistically insignificant
D-dimer values	3.48	-4.35, 11.31	16	0.38	Statistically insignificant
Procalcitonin	Could not be estimated				
Lactate dehydrogenase levels	Could not be estimated				
C-reactive protein	-29.22	-80.15, 21.71	15	0.26	Statistically insignificant

*Patient’s Characteristics*

**Fig. 2:**
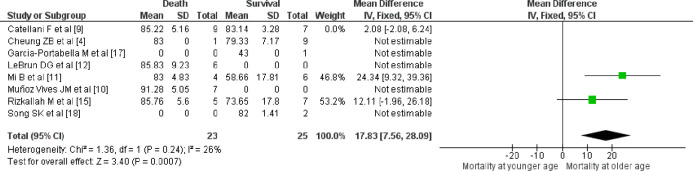
Forest plot suggesting significant association of older age with mortality in COVID-19 infected patients with fractures

Among the patient’s characteristics that considered patients’ age, gender, individual comorbidities and the total number of comorbidities, we found that the older age of the patients had a significant influence on increased mortality (*p*=0.0007, Table 3, Figure 2). The other associations were statistically insignificant (Tables 2 and 3).

*Fracture Characteristics*

**Fig. 3:**
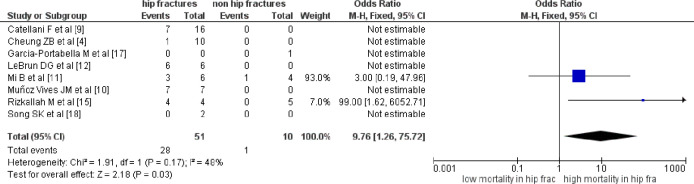
Forest plot suggesting significant association of hip fractures with mortality in COVID-19 infected patients with fractures

Concerning the fracture’s characteristics, we compared the mortality rates between hip and non-hip fractures, and between upper and lower limb fractures. Hip fractures were found to have significantly higher mortality rates (*p*=0.03, Table 2, Figure 3). The remaining associations were statistically insignificant (Table 2).

*Inflammatory Markers*

From the available data, we analyzed the association of inflammatory markers that consisted of total leucocyte counts (TLC), D-dimer values, Procalcitonin, Lactate Dehydrogenase levels, and CRP levels. While no statistically significant associations were observed with TLC, D-dimer values and CRP levels, the associations of Procalcitonin and Lactate dehydrogenase levels could not be analysed due to insufficient comparative data for meta-analysis (Table 3).

*Surgery Related Parameters*

Among the surgery-related parameters, we analyzed the mortality associated with operative management vs. non-operative management, ASA grades, delay in operative management, and hemiarthroplasty vs. cephalomedullary nailing for hip fractures. However, no statistically significant association was observed (Tables 2 and 3).

## Discussion

*What is known about mortality in COVID-19 patients?*

The mortality rates with COVID-19 infection are high. The earlier predicted overall death rate with COVID-19 infection was approximately 4% [19]. However, with further estimates, variable mortality has been observed in different regions of the world, ranging between 0.3% to 13.1% [20]. The disease is highly contagious and has now spread globally. The elderly population is at the higher risk of developing complications due to COVID-19 illness, as evident from the high mortality in older patients [21]. The mortality rates range between 8% to 16% in the 70-79 years’ age group and 15% to 26% in patients aged above 80 years. Besides age, the preexisting comorbidities especially cardiovascular and cerebrovascular disorders have higher mortality rates [22]. Multiple comorbidities can result in poor clinical outcomes, need for respiratory support and mechanical ventilation [23]. However, more evidence is still needed to establish the individual contribution of each comorbidity in COVID-19 related mortality.

*How Mortality Differs in COVID-19 Patients with Fractures?*

The current evidence concerning fracture management is limited in COVID-19 positive patients. The restrictions around the world with lockdown measures have markedly brought down the overall fracture incidence [16]. The patterns of fractures in the current scenario suggest a markedly higher proportion of low-energy injury trauma being the most common injury mechanism [24]. Also, the proportion of patients belonging to geriatric age groups has markedly increased which could possibly be attributed to osteoporosis and risk of falls. Considering the available evidence, there appears a substantially higher risk of complications and mortality in fracture patients with concomitant COVID-19 infection [4, 9-18]. However, these outcomes cannot be generalized for all age groups, considering that the larger proportion of patients belonged to the geriatric age group and those with comorbidities. Thus, fracture and old age could be possible contributors in predicting mortality. Additionally, the hip fractures formed the major chunk of injuries in the current pandemic situation which frequently required inpatient management. The quantitative analysis of our review supported the significant association of older age and hip fractures with significantly higher mortality. No significant association with individual comorbidities or the number of comorbidities could be established. The ASA grading, which is a well established prognostic tool for hip fractures in elderly patients [25], failed to show a statistical significance with the mortality rates in COVID-19 infected patients. A limited number of studies and volume of patients might have contributed to this discrepancy. The mortality rate in COVID-19 positive fracture patients is markedly high compared to non-fractured COVID-19 positive patients [15]. The early mortality in COVID-19 patients undergoing surgeries is estimated to be around 23·8% [26]. Our analysis suggests a higher mortality in COVID-19 patients undergoing fracture surgeries, especially hip fracture-related surgeries. Also, the mortality is way higher compared to the non-fractured COVID-19 patients. 

*Should Surgery Be Delayed in Fracture Patients?*

While there had been a general predilection towards delaying surgery, the lower limb fractures especially hip fractures should be prioritized considering the complications related to delayed fracture management. However, the COVID-19 management should be prioritized and the fracture management can be delayed in patients with symptomatic COVID-19 related illness [27]. Surgery contributes to early fracture stabilization, appropriate mobilization, improvement in physiological ventilation, and general comfort of the patient. Therefore, patients should continue to undergo surgical treatment if they are clinically fit. Non-operative treatment should be considered in older patients with fractures that can be managed non-operatively and for whom surgery can be delayed without major complications such as distal radial fractures [11]. Most patients without clinical symptoms of hypoxia can safely undergo early surgical intervention after appropriate medical optimization [4]. If a patient with a proximal femoral fracture is confirmed positive for COVID-19 and symptomatic, the response to COVID-19 management may be prioritized and management of the fracture may be delayed. 

*Current Consensus in Operative Management of Fractures in COVID-19 Patients*

The families of the COVID-19 positive fracture patients should be counseled regarding the significantly higher mortality and complications especially with hip fractures. COVID-19 infection causes an increase in perioperative mortality in proximal femoral fractures. All trauma patients who require surgery should undergo screening for COVID-19 to avoid unnecessary risk to the patient if the surgery can be delayed by a couple of weeks with minimal impact on outcome [6]. All efforts should be made to segregate the fracture management of COVID-19 positive patients away from the management of COVID-19 negative patients to prevent the risk of nosocomial COVID-19 infection [28]. A few articles had suggested delaying surgical intervention until the inflammatory phase has resolved [29]. However, the current review as well as the included reports could not find any significant association between inflammatory markers and the mortality rates in COVID-19 positive fracture patients. The current guidelines support to proceed with early surgical intervention when the peripheral oxygen saturation (pO2) of >90% and a body temperature of <38°C are maintained.

*Suggestions from the Current Review*

With very limited evidence available regarding the mortality in COVID-19 fractures affected patients, it is difficult to determine the prognosis of the patients with operative and non-operative management. No consensus has been established whether to delay the fracture surgery or not and what could be the possible outcomes. A knowledge about the factors influencing the mortality related outcomes in fracture management can help surgeons in choosing the appropriate management according to the patient characteristics and fracture patterns. Our review suggests significantly higher early mortality with increasing age and hip fractures in COVID-19 infected patients. However, more evidence is required to establish valid recommendations due to limited number of studies related to fracture management in such patients with wide confidence intervals in the meta-analysis (Table 2). 

*Limitations*

There were several limitations of this review. The review is based on a small number of observational studies performed over a short period of time. The quality of evidence belongs to level III and level IV studies. The samples sizes of the included studies were small. Not all the parameters influencing the mortality in COVID-19 positive fracture patients were studied in every reviewed study. Most of the fractures studied belonged to the geriatric hip fracture group, as such patients frequently require early inpatient care. The outcomes with upper limb fractures may vary, and can potentially change the overall results as well. Lastly, the mortality rates accounted for the inpatient mortality while the patients were still under observation. A further change in mortality rates can occur with post-discharge follow-ups. Nevertheless, the current review fulfills the purpose of a comprehensive analysis which is lacking in the current scenario. Additionally, further evidence with a longer time frame would be needed for a detailed review especially meta-analysis that could establish a stronger inference. 

## Conclusion

The mortality rates in COVID-19 positive patients with fractures are considerably higher compared to COVID-19 positive patients without fractures and to the COVID-19 negative fracture patients. The available evidence suggests that older age and hip fractures are the chief parameters associated with higher mortality rates in COVID-19 positive fracture patients. Early surgical intervention should be preferred in hip fractures among COVID-19 positive patients for general stabilization and improvement of respiratory function. 
